# Supplementary material for: Factors Associated with the Risk of Major Adverse Cardiovascular Events in Patients with Ankylosing Spondylitis: A Nationwide, Population-Based Case—Control Study
Source: Int J Environ Res Public Health. 2022 Mar 30;19(7):4098. doi: 10.3390/ijerph19074098 (PMC8998897; doi:10.3390/ijerph19074098)
Supplement: Supplementary file 1 [file ijerph-19-04098-s001.zip › ijerph-1629004-supplementary.pdf]

Supplementary material

Table S1 – The International Codes of Diseases–Ninth Revision Clinical Modification codes of the diseases and manifestations

Disease name	International Codes of Diseases–Ninth Revision Clinical Modification codes
Ankylosing spondylitis	720.0
Components of major adverse cardiovascular events	
Myocardial infarction	410.X, except for 410.X2
Ischaemic stroke	433-436, except for 433.X0 and 434.X0
Percutaneous coronary intervention, percutaneous transluminal coronary angioplasty, or stent	Procedure codes 00.66, 36.03, 36.06, 36.07, and 36.09
Coronary artery bypass graft	Procedure codes 36.1 and 36.2
Comorbidities	
Hypertension	401–405
Hyperlipidaemia receiving lipid-lowering agents	272
Diabetes mellitus	250
Chronic kidney disease	580–587
Heart failure	428
Valvular heart disease	093.2, 394-397, 424, 746.3-746.6
Chronic obstructive pulmonary disease	490-493, 496
Extra-articular manifestations	
Acute anterior uveitis	364.00–364.02, 364.04–364.05, 364.3
Psoriasis	6961
Inflammatory bowel disease	555, 556

Table S2 – Incidence of MACE in the identified population with AS (n = 42595, person-years = 245279)

Variables	Patients with events, n (%)	Total person-years	Incidence rate (95% CI) [1]
MACE	1151 (2.70)	245279	469.26 (469.18-469.35)
Male (n=27236)	766 (2.81)	158299	483.89 (483.79-484.00)
Female (n=15359)	385 (2.51)	86980	442.63 (442.49-442.77)
AS diagnosis at age < 65 years (n=38231)	612 (1.60)	222250	275.37 (275.30-275.43)
AS diagnosis at age ≥ 65 years (n=4364)	539 (12.35)	23029	2340.47 (2339.84-2341.09)
Myocardial infarction	241 (0.57)	248329	97.05 (97.01-97.09)
Male (n=27236)	179 (0.66)	160181	111.75 (111.70-111.80)
Female (n=15359)	62 (0.40)	88148	70.34 (70.28-70.39)
AS diagnosis at age < 65 years (n=38231)	142 (0.37)	223846	63.44 (63.40-63.47)
AS diagnosis at age ≥ 65 years (n=4364)	99 (2.27)	24483	404.36 (404.10-404.61)
Ischemic stroke	646 (1.52)	246934	261.61 (261.54-261.67)
Male (n=27236)	388 (1.42)	159534	243.21 (243.13-243.29)
Female (n=15359)	258 (1.68)	87400	295.19 (295.08-295.31)
AS diagnosis at age < 65 years (n=38231)	296 (0.77)	223300	132.56 (132.51-132.60)
AS diagnosis at age ≥ 65 years (n=4364)	350 (8.02)	23634	1480.93 (1480.44-1481.43)
Coronary artery bypass graft	53 (0.12)	248805	21.30 (21.28-21.32)
Male (n=27236)	43 (0.16)	160546	26.78 (26.76-26.81)

Female (n=15359)	10 (0.07)	88259	11.33 (11.31-11.35)
AS diagnosis at age < 65 years (n=38231)	33 (0.09)	224158	14.72 (14.71-14.74)
AS diagnosis at age ≥ 65 years (n=4364)	20 (0.46)	24647	81.14 (81.03-81.26)
Percutaneous coronary intervention	471 (1.11)	247467	190.33 (190.27-190.38)
Male (n=27236)	356 (1.31)	159558	223.12 (223.04-223.19)
Female (n=15359)	115 (0.75)	87909	130.82 (130.74-130.89)
AS diagnosis at age < 65 years (n=38231)	302 (0.79)	223308	135.24 (135.19-135.29)
AS diagnosis at age ≥ 65 years (n=4364)	169 (3.87)	24159	699.54 (699.21-699.88)

[1] per 100000 person-years. AS, ankylosing spondylitis. MACE, major adverse cardiovascular event.
